# Supplementary material for: The causal relationship between the human gut microbiota and pyogenic arthritis: a Mendelian randomization study
Source: Front Cell Infect Microbiol. 2024 Nov 26;14:1452480. doi: 10.3389/fcimb.2024.1452480 (PMC11629706; doi:10.3389/fcimb.2024.1452480)
Supplement: Supplementary file 1 [file DataSheet1.zip › mendelian test/ebi-a-GCST90027568.csv_leave_one_out.pdf]

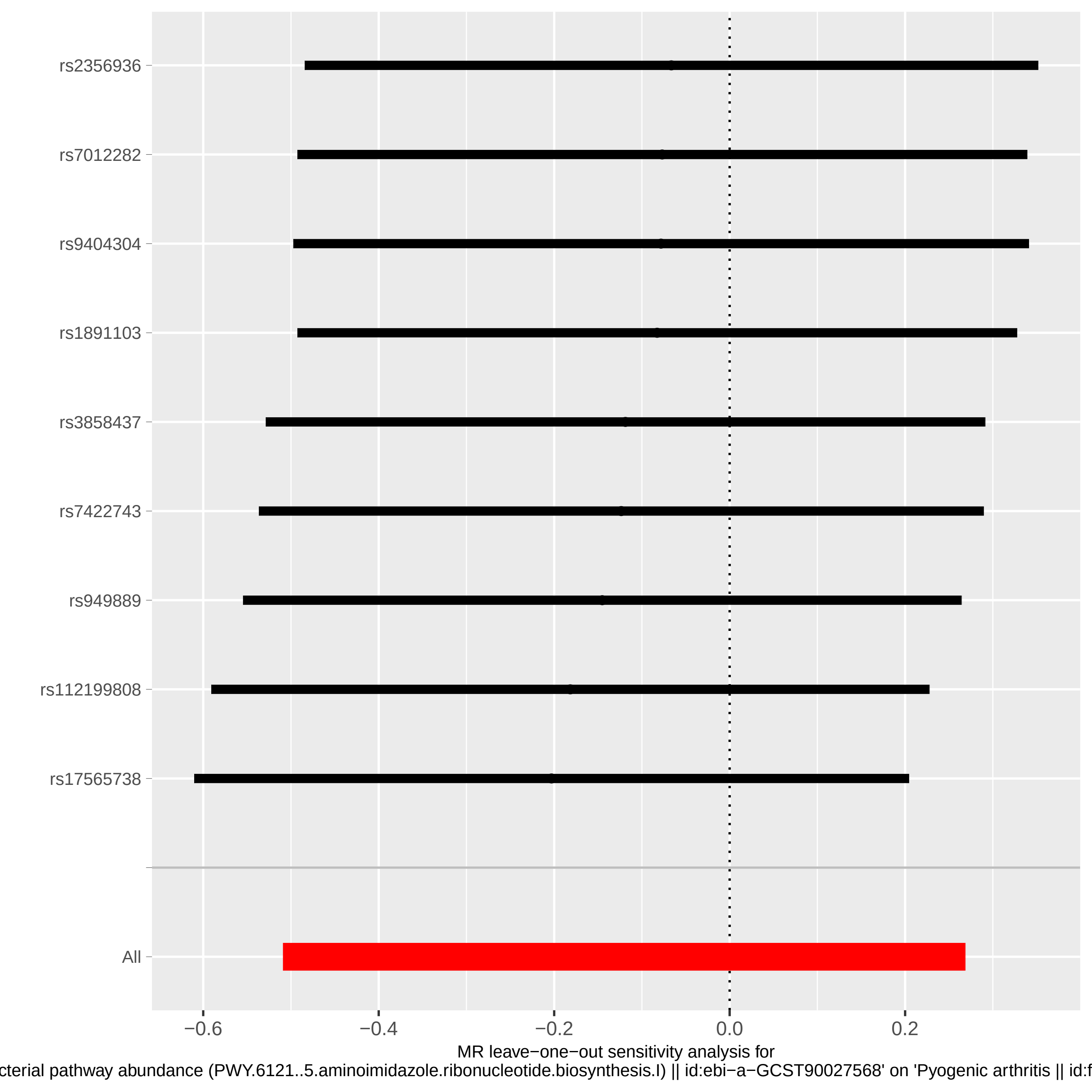
rs2356936
rs7012282
rs9404304
rs1891103
rs3858437
rs7422743
rs949889
rs112199808
rs17565738
All
−0.6
−0.4
−0.2
0.0
0.2
MR leave−one−out sensitivity analysis for
cterial pathway abundance (PWY.6121..5.aminoimidazole.ribonucleotide.biosynthesis.I) || id:ebi−a−GCST90027568' on 'Pyogenic arthritis || id:f
